# Symptomatic and preventive medication use according to age and frailty in Australian and Japanese nursing homes

**DOI:** 10.1007/s40520-023-02600-x

**Published:** 2023-11-07

**Authors:** Shin J. Liau, Shota Hamada, Agathe D. Jadczak, Nobuo Sakata, Samanta Lalic, Rumiko Tsuchiya-Ito, Reina Taguchi, Renuka Visvanathan, J. Simon Bell

**Affiliations:** 1 Centre for Medicine Use and Safety, Faculty of Pharmacy and Pharmaceutical Sciences, Monash University, Melbourne, Australia; 2 Research Department, Institute for Health Economics and Policy, Association for Health Economics Research and Social Insurance and Welfare, Tokyo, Japan; 3 Department of Home Care Medicine, Graduate School of Medicine, The University of Tokyo, Tokyo, Japan; 4 Department of Health Services Research, Institute of Medicine, University of Tsukuba, Tsukuba, Japan; 5 Adelaide Geriatrics Training and Research with Aged Care (GTRAC) Centre, Adelaide Medical School, Faculty of Health and Medical Sciences, University of Adelaide, Adelaide, Australia; 6 Heisei Medical Welfare Group Research Institute, Tokyo, Japan; 7 Pharmacy Department, Monash Health, Melbourne, Australia

**Keywords:** Aged, Frailty, Nursing home, Polypharmacy, Medication review, Deprescribing

## Abstract

**Objective:**

To investigate symptomatic and preventive medication use according to age and frailty in Australian and Japanese nursing homes (NHs).

**Methods:**

Secondary cross-sectional analyses of two prospective cohort studies involving 12 Australian NHs and four Japanese NHs. Frailty was measured using the FRAIL-NH scale (non-frail 0–2; frail 3–6; most-frail 7–14). Regular medications were classified as symptomatic or preventive based on published lists and expert consensus. Descriptive statistics were used to compare the prevalence and ratio of symptomatic to preventive medications.

**Results:**

Overall, 550 Australian residents (87.7 ± 7.3 years; 73.3% females) and 333 Japanese residents (86.5 ± 7.0 years; 73.3% females) were included. Australian residents used a higher mean number of medications than Japanese residents (9.8 ± 4.0 vs 7.7 ± 3.7, *p* < 0.0001). Australian residents used more preventive than symptomatic medications (5.5 ± 2.5 vs 4.3 ± 2.6, *p* < 0.0001), while Japanese residents used more symptomatic than preventive medications (4.7 ± 2.6 vs 3.0 ± 2.2, *p* < 0.0001). In Australia, symptomatic medications were more prevalent with increasing frailty (non-frail 3.4 ± 2.6; frail 4.0 ± 2.6; most-frail 4.8 ± 2.6, *p* < 0.0001) but less prevalent with age (< 80 years 5.0 ± 2.9; 80–89 years 4.4 ± 2.6; ≥ 90 years 3.9 ± 2.5, *p* = 0.0042); while preventive medications remained similar across age and frailty groups. In Japan, there was no significant difference in the mean number of symptomatic and preventive medications irrespective of age and frailty.

**Conclusions:**

The ratio of symptomatic to preventive medications was higher with increasing frailty but lower with age in Australia; whereas in Japan, the ratio remained consistent across age and frailty groups. Preventive medications remained prevalent in most-frail residents in both cohorts, albeit at lower levels in Japan.

**Supplementary Information:**

The online version contains supplementary material available at 10.1007/s40520-023-02600-x.

## Introduction

Frailty is a state of reduced physiological reserve characterized by an increased vulnerability to medication-related harm and adverse outcomes including falls, delirium, and hospitalization [1]. Frailty may act as an effect modifier by modifying the risks and benefits of chronic medications [2]. It is estimated that over three quarters of residents in nursing homes (NHs) are frail [3]. Prescribing in this population is often guided by recommendations from disease-specific clinical guidelines that are based on research conducted in robust adults [2, 4, 5]. These recommendations are often extrapolated to NH settings despite residents being underrepresented or excluded from clinical trials [6]. This may expose residents to potential medication-related harm [2]. Prescribing in this setting is further complicated by the presence of complex multimorbidity and polypharmacy [1, 5].

A systematic review reported that residents of NHs received an average of 3.8–16.6 medications daily with a wide range in prevalence of symptomatic and preventive medications [7]. Gastrointestinal agents, diuretics, and analgesics were among the most prescribed medications [7]. As frailty progresses, residents’ goals of care may shift from chronic disease prevention to prioritize comfort-oriented care with management of symptoms to maintain quality of life [2, 8]. Optimizing medication management in this population involves balancing the use of symptomatic and preventive medications [2, 6]. There is increasing interest in frailty screening to identify residents who are most susceptible to adverse drug events and may benefit from a medication review [1, 5]. Incorporating frailty screening into medication review is consistent with international guidelines that recommend regular reassessment of the risk-to-benefit profile of individual regimens [1, 4, 5].

Existing studies examining patterns of symptomatic and preventive medication use have focused on people with advanced dementia in hospital and palliative care settings [9–11]. A European cross-sectional study of 4121 residents found that frailty was associated with higher prevalence of symptomatic medications (e.g., laxatives, analgesics), while preventive medications (e.g., bisphosphonates, vitamin D) were more prevalent among non-frail residents [12]. Despite recognition that transitions in frailty status may influence prescribing decisions, there is a lack of non-European studies comparing symptomatic and preventive medication use in the context of frailty. The objective of this study was to investigate the use of symptomatic and preventive medications according to age and frailty among residents of Australian and Japanese NHs.

## Methods

### Study design

This was a secondary cross-sectional analyses of baseline data collected from two independent prospective cohort studies in Australia and Japan. Both countries have an aging population for which optimizing medication management in long-term care has been identified as a policy priority [13, 14].

### Settings

Residential aged care services (RACS) in Australia, synonymous with NHs or long-term care facilities (LTCFs) in other countries, provide care and supported accommodation for people who can no longer live independently at home [15]. Medications are prescribed by general practitioners (GPs) who attend the RACS periodically but are not directly employed by aged care providers [15]. Medications are dispensed and packed by off-site community pharmacists into dose administration aids (e.g., multi-dose drug dispensing, blister packs) to facilitate administration by credentialed aged care staff [15].

Special NHs or *Tokuyo* in Japan are similar to RACS in Australia (i.e., permanent residence for adults with high long-term care needs). In contrast to Australia, medications are prescribed by primary care physicians who are contracted by the NHs. Community pharmacists dispense the medications and nursing staff manage the administration of medications. Medication cost is subsidized for residents in both countries.

### Data collection

In Australia, data were collected as part of the Frailty In Residential Sector over Time (FIRST) study, a prospective cohort study involving 12 RACS in South Australia [16]. All 12 RACS were operated by a large not-for-profit aged care provider. Residents staying for eight weeks or more were eligible to participate. Residents with a life expectancy of less than three months and those not capable of completing baseline assessments in English were excluded. Baseline data were collected from March to October 2019 by trained registered nurses. Comorbidities were based on documented diagnoses drawn from resident records. Activities of daily living (ADL) was assessed using Katz ADL [17]. Cognitive status was assessed using the Dementia Severity Rating Scale (DSRS) irrespective of dementia diagnosis [18]. Supplementary Table 1 details the full description of the scales. Of the 561 Australian residents included in the cohort study, 550 residents completed baseline assessments. The cohort was comparable to the average Australian NH population in terms of age (88 vs 85 years) and sex (73% vs 66%) [19].

In Japan, data were collected from a prospective cohort study involving four NHs located in Tokyo and Kanagawa prefecture. All four NHs were serviced by a large hospital and LTCF group. Residents were invited by NH staff to participate in the study. Residents receiving medications from other hospitals or clinics, and those with incomplete medication records were excluded. Baseline data were collected from October to December 2020 by trained NH staff. Comorbidities were based on documented diagnoses drawn from resident records. ADL was assessed using the Japanese *"Independence in Daily Living in Older People with Disabilities"* scale [20]. Cognitive status was assessed using the Japanese *"Independence in Daily Living in Older People with Dementia"* scale [21]. Of the 372 Japanese residents included in the cohort study, 333 residents completed baseline assessments. The cohort was comparable to the average Japanese NH population in terms of age (87 vs 86 years) and sex (73% vs 78%) [22].

### Frailty assessment

Frailty status was assessed using the FRAIL-NH scale in both Australia (English version) [23] and Japan (Japanese version) [24]. FRAIL-NH has been previously translated and back-translated into Japanese [24]; both versions are identical apart from the language [3]. Both the Australian and Japanese cohorts used the same seven FRAIL-NH domains (fatigue, resistance, ambulation, incontinence, loss of weight, nutritional approach, and help with dressing) to calculate residents’ frailty scores. Each domain is assigned a score of 0–2 points, with a total score ranging from 0 to 14. FRAIL-NH has similar predictive properties to Fried’s phenotype and Frailty Index [3]. The scale has been shown to be predictive of adverse outcomes including falls, hospitalization, and mortality among residents of NHs [3]. The same frailty categories were used for both cohorts: non-frail (0–2); frail (3–6); and most-frail (7–14). These cut-offs were previously established using receiver operating characteristic curves to determine the FRAIL-NH score that maximized sensitivity and specificity in predicting frailty based on Frailty Index [16]. Using the same FRAIL-NH domains and cut-offs in Australia and Japan facilitated comparison between cohorts and frailty groups.

### Medications

Data on all regular medications including oral, inhaled, topical, parenteral, dietary, and herbal supplements were extracted. The following data were recorded: active ingredient, strength, dose, frequency, and formulation. In Australia, data were extracted from residents’ regular medication charts which provide the most current medication administration records. The overall quality and accuracy of data entries were individually reviewed and evaluated by the research team. In Japan, medication dispensing data were extracted from electronic health records. Medications were coded according to their Anatomical Therapeutic Chemical (ATC) classifications [43]. Different strengths of the same medication, or combination medications that share a single ATC code were counted as one medication. Pro re nata (PRN) or as needed medications were not included.

Medications were categorized as symptomatic or preventive based on published lists and international expert consensus among four clinical pharmacists, two geriatricians, and a pharmacoepidemiologist [9, 10, 25, 26]. All medications were categorized using the same consensus list (Supplementary Table 2). Symptomatic medications were defined as medications predominantly used for comfort or symptom control (including medications used for the prophylaxis of short-term or acute events not associated with death [e.g., urinary tract infection]). Preventive medications were defined as medications predominantly used for long-term prevention or worsening of a disease or condition (including medications used for primary or secondary prevention of death/events associated with death [e.g., stroke]).

### Statistical analysis

Resident characteristics were presented as frequencies with percentages and means with standard deviations (SD). Categorical variables were compared using chi-square tests and continuous variables were compared using independent *t*-tests. Descriptive statistics were used to compare the prevalence and ratio of symptomatic to preventive medications stratified by age and frailty. One-way analysis of variance (ANOVA) was used to determine differences in mean number of medications between age and frailty groups. We performed a sensitivity analysis by excluding Japanese residents who died within three months of baseline data collection. This was because the Australian cohort did not include residents with an estimated life expectancy of less than three months. Statistical significance was set at *p* < 0.05. Analyses were performed using SAS 9.4 (SAS Institute Inc., Cary, NC) in Australia and Stata 17 (Stata Corp., College Station, TX) in Japan.

### Ethical considerations

The Australian study was approved by the University of Adelaide Human Research Ethics Committee (H-2018-247), South Australian Department for Health and Wellbeing Human Research Ethics Committee (HREC/20/SAH/15), Department of Human Services External Request Evaluation Committee (EREC/RMS0432), and Monash University Human Research Ethics Committee (23620). The Japanese study was approved by the ethical review board of the Institute for Health Economics and Policy, Japan (R2-002). Written informed consent was obtained from all residents or their next-of-kin.

## Results

In total, 550 Australian residents (87.7 ± 7.3 years; 73.3% female) and 333 Japanese residents (86.5 ± 7.0 years; 73.3% female) were included in the data analysis (Table 1). The mean FRAIL-NH scores were 6.3 ± 3.2 in Australia and 7.3 ± 3.6 in Japan (*p* < 0.0001). Both Australia and Japan had similar proportions of non-frail residents (12.4% vs 12.9%). In Australia, the proportion of frail and most-frail residents were evenly distributed (42.0% vs 45.6%); whereas in Japan, there were considerably more residents who were most-frail than frail (61.9% vs 25.2%). In Australia, there was a higher proportion of most-frail residents among those aged < 80 years (57.9%) compared to those aged 80–89 years (42.5%) and ≥ 90 years (44.7%) (Supplementary Fig. 1). Prevalence of dementia was similar across age groups (34.0–39.5%) but was higher among most-frail (48.8%) compared to non-frail (20.6%) and frail (28.8%) residents in the Australian cohort. In Japan, the proportion of most-frail residents ranged from 57.8 to 65.9% across age groups. Prevalence of dementia was higher with age (55.6% in < 80 years; 70.3% in 80–89 years; 74.8% in ≥ 90 years) and frailty (55.8% in non-frail; 64.3% in frail; 75.2% in most-frail) in the Japanese cohort.

**Table 1:** Baseline characteristics of residents

Characteristics	Australia (*n* = 550)	Japan (*n* = 333)	*p*-value
Age, mean (SD)	87.7 (7.3)	86.5 (7.0)	0.02
< 80 years, *n* (%)	76 (13.8)	45 (13.5)	
80–89 years, *n* (%)	228 (41.5)	165 (49.5)	
≥ 90 years, *n* (%)	246 (44.7)	123 (36.9)	
Sex, *n* (%)			1.00
Female	403 (73.3)	244 (73.3)	
Male	147 (26.7)	89 (26.7)	
Comorbidities, *n* (%)^a^			
Coronary heart disease	155 (28.3)	39 (11.7)	< 0.0001
Stroke	161 (29.4)	118 (35.4)	0.06
Dementia	202 (36.9)	233 (70.0)	< 0.0001
Diabetes	131 (23.9)	74 (22.2)	0.59
Polypharmacy (≥ 9 meds), *n* (%)	332 (60.4)	137 (41.1)	< 0.0001
No. of medications, mean (SD)	9.8 (4.0)	7.7 (3.7)	< 0.0001
Symptomatic	4.3 (2.6)	4.7 (2.6)	0.01
Preventive	5.5 (2.5)	3.0 (2.2)	< 0.0001
FRAIL-NH score, mean (SD)	6.3 (3.2)	7.3 (3.6)	< 0.0001
Non-frail (0–2), *n* (%)	68 (12.4)	43 (12.9)	
Frail (3–6), *n* (%)	231 (42.0)	84 (25.2)	
Most-frail (7–14), *n* (%)	251 (45.6)	206 (61.9)	
Activities of daily living (ADL), *n* (%)^b,c^			
Moderately/fully independent	7 (1.3)	7 (2.1)	N/A
Not independent	543 (98.7)	326 (97.9)	N/A
Cognitive status, *n* (%)^d^^,e^			
No/minimal impairment	133 (24.2)	102 (30.6)	N/A
Cognitive impairment	417 (75.8)	231 (69.4)	N/A

*N/A* not applicable; *SD* standard deviation

Full description of scales available in Supplementary Table 1

^a^Australia: data available for *n* = 547 only

^b^Australia: Katz ADL (Not independent 0–2; Moderately/fully independent 3–6)

^c^Japan: *"Independence in Daily Living in Older People with Disabilities"﻿ [Japanese original scale]* (Not independent: Ranks A, B and C; Moderately/fully independent: Rank J)

^d^Australia: Dementia Severity Rating Scale (DSRS) (No/minimal impairment 0–11; Cognitive impairment 12–54)

^e^Japan: *"Independence in Daily Living in Older People with Dementia" [Japanese original scale]* (No impairment: Independent, Ranks I and II; Cognitive impairment: Ranks III, IV and M)

**Fig. 1:**
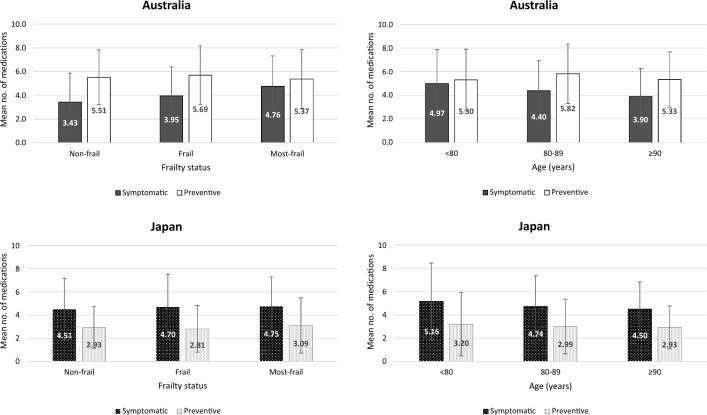
Mean number of symptomatic and preventive medications according to frailty status and age

Overall, Australian residents used a higher mean number of medications than Japanese residents (9.8 ± 4.0 vs 7.7 ± 3.7, *p* < 0.0001). Australian residents used more preventive than symptomatic medications (5.5 ± 2.5 vs 4.3 ± 2.6, *p* < 0.0001), while Japanese residents used more symptomatic than preventive medications (4.7 ± 2.6 vs 3.0 ± 2.2, *p* < 0.0001). In Australia, the mean number of symptomatic medications was higher with increasing frailty (non-frail 3.4 ± 2.6; frail 4.0 ± 2.6; most-frail 4.8 ± 2.6, *p* < 0.0001) but lower with age (< 80 years 5.0 ± 2.9; 80–89 years 4.4 ± 2.6; ≥ 90 years 3.9 ± 2.5, *p* = 0.0042), while preventive medications remained similar across age and frailty groups (Supplementary Table 3a). This corresponded to a higher ratio of symptomatic to preventive medications among frailer Australian residents (non-frail 0.62; frail 0.69; most-frail 0.89) (Fig. 1), but a lower ratio with age (< 80 years 0.94; 80–89 years 0.76; ≥ 90 years 0.73). In Japan, there was no significant difference in the mean number of symptomatic and preventive medications across age and frailty groups. Consequently, the ratio of symptomatic to preventive medications was largely consistent across age and frailty groups in Japan (1.54–1.67).

### Symptomatic medications

The most prevalent medication classes stratified by age and frailty are presented in Table 2. Among symptomatic medications, paracetamol (69.1%), proton pump inhibitors (PPIs) (46.0%), and contact laxatives (32.9%) were most prevalent in Australia; while osmotic laxatives (56.5%), PPIs (44.4%), and contact laxatives (33.9%) were most prevalent in Japan. The top five medications within each country were similar across age and frailty groups although variations in the prevalence were observed. Paracetamol use was more prevalent among frailer Australian residents (non-frail 60.3%, frail 63.6%, most-frail 76.5%), while the overall prevalence among Japanese residents was only 3.6%. PPIs were most prevalent among non-frail (48.5%) compared to frail (47.2%) and most-frail (44.2%) Australian residents; whereas the opposite was observed in Japan (non-frail 34.9%; frail 33.3%; most-frail 51.0%). Buprenorphine patches and pregabalin were only prevalent in non-frail Australian residents, while benzodiazepines were only prevalent in Australian residents > 80 years. *Yokukansan* (Japanese herbal medication [*Kampo*] commonly used for behavioral and psychological symptoms of dementia) [27] was only prevalent in non-frail Japanese residents, while Z-drugs were only prevalent in frail Japanese residents.

**Table 2:** Most prevalent medication classes stratified by frailty status and age

	Medications (Prevalence, %)	
	Australia	Japan
*Symptomatic*		
Overall	Paracetamol (69.1)	Osmotic laxative (56.5)
	PPI (46.0)	PPI (44.4)
	Contact laxative (32.9)	Contact laxative (33.9)
	Ocular lubricant (27.3)	White petrolatum (32.7)
	Osmotic laxative (26.5)	Topical heparinoid (27.9)
*Frailty status*		
Non-Frail	Paracetamol (60.3)	Osmotic laxative (65.1)
	PPI (48.5)	PPI; Contact laxative (34.9)
	Ocular lubricant (23.5)	White petrolatum (32.6)
	Contact laxative (20.6)	Topical heparinoid (30.2)
	Buprenorphine patch; Pregabalin & derivatives^a^ (14.7)	*Yokukansan* (14.0)
Frail	Paracetamol (63.6)	Osmotic laxative (56.0)
	PPI (47.2)	Topical heparinoid (38.1)
	Ocular lubricant (25.1)	PPI; Contact laxative (33.3)
	Contact laxative (23.4)	White petrolatum (29.8)
	Osmotic laxative (21.6)	Z-drug (16.7)
Most-frail	Paracetamol (76.5)	Osmotic laxative (54.9)
	Contact laxative (45.0)	PPI (51.0)
	PPI (44.2)	Contact laxative; White petrolatum (34.0)
	Osmotic laxative (36.3)	Topical heparinoid (23.3)
	Ocular lubricant (30.3)	GI stimulant; Probiotic (17.5)
*Age (years)*		
< 80	Paracetamol (68.4)	Osmotic laxative (46.7)
	PPI (47.4)	PPI (44.4)
	Contact laxative (34.2)	White petrolatum (40.0)
	Osmotic laxative (32.9)	Topical heparinoid (35.6)
	Benzodiazepine (25.0)	Contact laxative (20.0)
80–89	Paracetamol (67.5)	Osmotic laxative (59.4)
	PPI (51.3)	PPI (43.6)
	Contact laxative (34.6)	Contact laxative; White petrolatum (33.3)
	Ocular lubricant (25.9)	Topical heparinoid (29.1)
	Osmotic laxative (21.9)	GI stimulant; Probiotic (14.5)
≥ 90	Paracetamol (70.7)	Osmotic laxative (56.1)
	PPI (40.7)	PPI (45.5)
	Contact laxative (30.9)	Contact laxative (39.8)
	Ocular lubricant (30.1)	White petrolatum (29.3)
	Osmotic laxative (28.9)	Topical heparinoid (23.6)

	Medications (Prevalence, %)	
	Australia	Japan
*Preventive*		
Overall	Vitamin D (52.0)	CCB (36.6)
	Antiplatelet (40.0)	Antiplatelet (24.3)
	Statin (33.6)	Loop diuretic (19.2)
	Loop diuretic (31.1)	Anticholinesterase (17.4)
	Beta-blocker (26.2)	ARB (17.1)
*Frailty status*		
Non-Frail	Statin (52.9)	CCB (37.2)
	Vitamin D (47.1)	Antiplatelet (20.9)
	Antiplatelet (38.2)	Thyroid hormone (16.3)
	Beta-blocker (32.4)	Statin; Loop diuretic (14.0)
	Loop diuretic (30.9)	Vitamin D; Vitamin B12; Xanthine oxidase inhibitor (11.6)
Frail	Vitamin D (50.2)	CCB (41.7)
	Antiplatelet (44.2)	Antiplatelet (22.6)
	Statin (36.8)	Anticholinesterase; ARB (19.0)
	Loop diuretic (34.2)	Potassium (14.3)
	Beta-blocker (28.6)	Loop diuretic (13.1)
Most-frail	Vitamin D (55.0)	CCB (34.5)
	Antiplatelet (36.7)	Antiplatelet (25.7)
	Loop diuretic (28.3)	Loop diuretic (22.8)
	Statin (25.5)	Anticholinesterase (18.4)
	SSRI (23.9)	ARB (18.0)
*Age (years)*		
< 80	Vitamin D (61.8)	Antiplatelet (31.1)
	Statin (44.7)	CCB (26.7)
	Antiplatelet (36.8)	Anticholinesterase; ARB (17.8)
	SNRI and mirtazapine (23.7)	Direct factor Xa inhibitor; Valproate (15.6)
	Loop diuretic; SSRI (21.1)	Statin (13.3)
80–89	Vitamin D (46.5)	CCB (38.8)
	Antiplatelet (40.8)	Antiplatelet (22.4)
	Statin (39.9)	Loop diuretic (16.4)
	Loop diuretic (32.5)	Anticholinesterase; ARB (15.8)
	Beta-blocker (29.8)	Statin (11.5)
≥ 90	Vitamin D (54.1)	CCB (37.4)
	Antiplatelet (40.2)	Loop diuretic (27.6)
	Loop diuretic (32.9)	Antiplatelet (24.4)
	Beta-blocker (27.6)	Anticholinesterase (19.5)
	Statin (24.4)	ARB (18.7)

^a^Derivatives include levetiracetam, lamotrigine, topiramate (N03AX); ARB, angiotensin II receptor blocker (C09CA); CCB, dihydropyridine calcium channel blocker (C08CA); GI stimulant, gastrointestinal stimulant (A03FA); PPI, proton pump inhibitor (A02BC); SNRI, selective noradrenaline reuptake inhibitor (N06AX); SSRI, selective serotonin reuptake inhibitor (N06AB); *Yokukansan*, traditional Japanese herbal medication for changed behaviors. Full list of ATC codes used available in Supplementary Table 4

### Preventive medications

Among preventive medications, vitamin D supplements (52.0%), antiplatelets (40.0%), and statins (33.6%) were most prevalent in Australia; while dihydropyridine calcium channel blockers (CCBs) (36.6%), antiplatelets (24.3%), and loop diuretics (19.2%) were most prevalent in Japan. In Australia, a consistent pattern was observed whereby statins, vitamin D, antiplatelets, and loop diuretics were among the top preventive medications across age and frailty groups. In contrast, the overall prevalence of statins and vitamin D in Japan was only 9.4% and 8.1%, respectively. In Japan, a consistent pattern was also observed whereby CCBs, antiplatelets, and loop diuretics were among the top preventive medications across age and frailty groups. Japanese residents were more commonly prescribed CCBs, while Australian residents were more commonly prescribed beta-blockers. Anticholinesterase use was only prevalent among frail (19.0%) and most-frail (18.4%) Japanese residents. Conversely, overall prevalence of anticholinesterase use (8.0%) was minimal in Australia.

### Sensitivity analysis

In the sensitivity analysis excluding 10 (3.0%) Japanese residents who died within three months of baseline data collection, there remained no significant difference in the mean number of symptomatic and preventive medication use in all age and frailty groups (Supplementary Table 3b). The ratio of symptomatic to preventive medications in the adjusted analysis (1.52–1.67) was similar to the main analysis (1.54–1.67) across age and frailty groups in Japan.

## Discussion

This is the first study to compare symptomatic and preventive medication use according to age and frailty in Australian and Japanese NHs. Overall, Australian residents used a higher mean number of medications than Japanese residents. Australian residents used more preventive than symptomatic medications, while the opposite was observed for Japanese residents. Preventive medications were prevalent across all age and frailty groups in both cohorts. The ratio of symptomatic to preventive medications was higher with increasing frailty in Australia but was consistent irrespective of frailty status in Japan.

In Australia, symptomatic medication use was more prevalent with higher frailty levels but less prevalent with advanced age; whereas in Japan, symptomatic medication use was similar across age and frailty groups. Australian findings were in line with a European study that reported a higher prevalence of symptomatic medications among residents who were frail compared to non-frail [12]. This may be because clinicians gave greater consideration to frailty than chronological age in their treatment decision-making. Clinicians may prioritize symptom control over intensive treatment of chronic diseases in frail and most-frail residents. While frailty is characterized by an age-related decline in physiological systems, frailty and age are not synonymous [1, 2]. Frailty may be a better predictor of medication response than chronological age [2, 6]. Interestingly, the Australian cohort had a lower proportion of most-frail residents in those ≥ 90 years compared to younger age groups. Prevalence of dementia was lower in Australian residents ≥ 90 years when compared across age groups, but higher in most-frail residents when compared across frailty groups. Previous studies have reported high symptom burden including pain, constipation, and fatigue among residents with dementia [28, 29]. This may lead to increased use of symptomatic medications to manage symptom burden, which may partly explain the higher ratio of symptomatic to preventive medication use in the Australian cohort with increasing frailty but not with older age. In the Japanese cohort, the higher use of symptomatic over preventive medications across all age and frailty groups may also be attributable to the higher overall prevalence of dementia.

There was no significant difference in the mean number of preventive medications across age and frailty groups in both cohorts. This contrasted with the European findings that reported a lower number of preventive medications among frail compared to non-frail residents [12]. Among the top five preventive medication classes in the Australian and Japanese cohorts, four were cardiovascular medications. The higher prevalence of antiplatelets, statins, and loop diuretics in Australia compared to Japan likely reflects the higher prevalence of coronary heart disease in the cohort (Australia 28.3%; Japan 11.7%). Japanese residents were more likely to be prescribed CCBs partly due to the lower risks of adverse events [30, 31]. Japanese residents also had a considerably lower prevalence of vitamin D use (Japan 8.1%; Australia 52.0%), which may reflect initiatives in Australian NHs to increase vitamin D supplementation [32]. Prevalence of anticholinesterase use was low in Australia (8.0%) but high in Japan (17.4%). This may be partly explained by the lower prevalence of documented dementia diagnoses in the Australian (36.9%) compared to the Japanese cohort (70.0%).

There has been increasing interest among some Australian and Japanese clinicians in deprescribing [33–35]. The lower prevalence of statins among most-frail compared to non-frail and frail residents in both cohorts may partly be due to deprescribing upon reassessment of the individual risk-to-benefit profile. Residents with advanced frailty often have limited life expectancy and may have a lower likelihood of achieving maximum therapeutic benefit from long-term preventive treatments [2, 8]. Our findings suggest a potential opportunity to deprescribe chronic medications for which potential harms outweigh benefits among frail residents. However, existing disease-specific clinical guidelines rarely recommend how or when to deprescribe medications that are unnecessary or no longer appropriate [36]. This is despite evidence of potential over-treatment of chronic conditions such as hypertension and diabetes in long-term care settings [37, 38]. Tools such as the Screening Tool of Older Persons Prescriptions in Frail adults with limited life expectancy (STOPPFrail) [39] and the Screening Tool for Older Person’s Appropriate Prescriptions for Japanese (STOPP-J) [40] criteria list medications that may be suitable for deprescribing.

There may be greater opportunity to deprescribe preventive medications in Australia than in Japan. It is not clear whether Japanese residents had preventive medications deprescribed at or prior to NH admission, or to what extent the least frail Japanese residents may benefit from medication continuation. Qualitative focus groups and interviews with Australian and Japanese healthcare professionals would be worthwhile to explore possible reasons for the differences observed. Recent position statements emphasize the value of medication review to ensure alignment of regimens to residents’ goals of care [1, 5, 6]. Frailty screening may complement the use of approaches such as the Supportive and Palliative Care Indicators Tool (SPICT) in identifying residents for review [41]. Overall, 21.5% of Australian residents received a pharmacist-led medication review within 90 days of NH admission [42]. Moving forward, comprehensive medication reviews may focus on individualizing treatment based on residents’ frailty status and changing goals of care.

### Strengths and limitations

Medication data were sourced from dispensing and administration records, providing an accurate reflection of residents’ actual medication use compared to prescription records. Both cohorts used the same seven FRAIL-NH domains and cut-off scores for frailty assessment. Validated tools were used to describe baseline characteristics although different tools were used to measure ADL and cognitive status in the two cohorts. The comparability of both cohorts was established with similarities in age, sex, and prevalence of diabetes. However, differences were observed in the documented prevalence of dementia diagnoses and coronary heart disease. Both cohorts were comparable to the respective national average NH population in terms of age and sex. The sample size in both cohorts was relatively small; therefore, it was not possible to generalize the patterns of medication use to all Australian and Japanese residents. Some residents who were most-frail may have been omitted from the Australian cohort as those with a life expectancy of less than three months were excluded.

Similar to other studies utilizing medication data, information on treatment indication was not available and so it was not possible to assess the clinical appropriateness of medication regimens for individual residents. A comprehensive and holistic evaluation at the resident level would be required to determine the appropriateness of individual symptomatic and preventive medications. Some medications may be used for both symptomatic and preventive purposes. Therefore, the medication categorization was an approximation based on multidisciplinary consensus and previously published lists. It is not clear to what extent the pattern of medication use in each country may reflect underlying cultural predispositions to specific chronic conditions. Given that the analyses were cross-sectional, it was not possible to determine whether the lower prevalence of some chronic medications (e.g., statins) were due to deprescribing among frailer residents. Some preventive medications may indeed be necessary in frail residents.

## Conclusions

Australian residents used more preventive than symptomatic medications, while Japanese residents used more symptomatic than preventive medications. The ratio of symptomatic to preventive medications was higher with increasing frailty but lower with age in Australia; whereas in Japan, the ratio remained consistent across age and frailty groups. Preventive medications remained prevalent in most-frail residents in both cohorts, albeit at lower levels in Japan. Identification of frail residents may assist with targeting initiatives to deprescribe long-term preventive medications for which potential harms outweigh benefits, while assessing the need for medications to manage symptoms and optimize quality of life.

### Supplementary Information

Below is the link to the electronic supplementary material.

Supplementary file1 (PDF 415 KB)

## Data Availability

The authors confirm that all relevant data are included in the article and supplementary file.
