# Supplementary material for: Symptomatic and preventive medication use according to age and frailty in Australian and Japanese nursing homes
Source: Aging Clin Exp Res. 2023 Nov 7;35(12):3047–57. doi: 10.1007/s40520-023-02600-x (PMC10721681; doi:10.1007/s40520-023-02600-x)
Supplement: Supplementary file 1 — Supplementary file1 (PDF 415 KB) [file 40520_2023_2600_MOESM1_ESM.pdf]

SUPPLEMENTARY MATERIAL

Symptomatic and Preventive Medication Use According to Age and Frailty in Australian and Japanese Nursing Homes

Shin J. Liau[1], Shota Hamada[2,3,4], Agathe D. Jadczak[5], Nobuo Sakata[4,6], Samanta Lalic[1,7], Rumiko Tsuchiya-Ito[2], Reina Taguchi[2], Renuka Visvanathan[5], J. Simon Bell[1]

[1] Centre for Medicine Use and Safety, Faculty of Pharmacy and Pharmaceutical Sciences, Monash University, Melbourne, Australia

[2] Research Department, Institute for Health Economics and Policy, Association for Health Economics Research and Social Insurance and Welfare, Tokyo, Japan

[3] Department of Home Care Medicine, Graduate School of Medicine, The University of Tokyo, Tokyo, Japan

[4] Department of Health Services Research, Institute of Medicine, University of Tsukuba, Tsukuba, Japan

[5] Adelaide Geriatrics Training and Research with Aged Care (GTRAC) Centre, Adelaide Medical School, Faculty of Health and Medical Sciences, University of Adelaide, Adelaide, Australia

[6] Heisei Medical Welfare Group Research Institute, Tokyo, Japan

[7] Pharmacy Department, Monash Health, Melbourne, Australia

Corresponding author: Shin J. Liau (Shin.Liau@monash.edu)

**Appendix**	**Page**
**Supplementary Table 1.**<br>Full description of activities of daily living (ADL) and cognitive scales	2
**Supplementary Table 2.**<br>List of symptomatic and preventive medication classifications	3
**Supplementary Table 3a.**<br>Mean number of symptomatic and preventive medications according to age and frailty	10
**Supplementary Table 3b.**<br>Sensitivity analysis excluding Japanese residents who died within three months of baseline data collection	10
**Supplementary Table 4.**<br>List of Anatomical Therapeutic Chemical (ATC) codes for medication classes	11
**Supplementary Figure 1.**<br>Proportion of residents stratified by age, frailty, and dementia status	12

Supplementary Table 1. Full description of activities of daily living (ADL) and cognitive scales

Activities of daily living (ADL)		n	(%)
**Australia: Katz ADL**			
Moderately/fully independent	Scores 5-6: Fully Independent	0	0.0
	Scores 3-4: Moderately Independent	7	1.3
Not independent	Scores 0-2: Not Independent	543	98.7
**Japan: "*Independence in Daily Living in Older People with Disabilities*" *[Japanese original scale]***			
Moderately/fully independent	Rank J: Some disabilities, but daily living is mostly independent, capable of going outdoors unassisted	7	2.1
Not independent	Rank A: Indoor living predominantly independent, but unable to go out without assistance	79	23.7
	Rank B: Some assistance needed for indoor living, also lies in bed for much of the daytime, although sitting position is possible	181	54.4
	Rank C: Bedridden all day, requires assistance with excretion/urination, meals, and dressing/undressing	66	19.8
**Cognitive status**		n	%
**Australia: Dementia Severity Rating Scale (DSRS)**			
No/minimal impairment	Scores 0-11: No dementia	133	24.2
Cognitive impairment	Scores 12-18: Mild dementia	96	17.5
	Scores 19-36: Moderate dementia	195	35.5
	Scores 37-54: Severe dementia	126	22.9
**Japan: "*Independence in Daily Living in Older People with Dementia*" *[Japanese original scale]***			
No impairment	Independent	5	1.5
	Rank I: Has some type of dementia, but almost independent in terms of daily living at home and in society	18	5.4
	Rank II: Some daily life-disturbing symptoms, behaviours and problems in communication seen but can lead daily life independently if kept watched by someone	79	23.7
Cognitive impairment	Rank III: Daily life-disturbing symptoms, behaviours, and problems in communication that require assistance	112	33.6
	Rank IV: Daily life-disturbing symptoms, behaviours, and problems in communication frequently require assistance	110	33.0
	Rank M: Marked psychiatric symptoms/related symptoms or serious physical disorders require expert management	9	2.7

Supplementary Table 2. List of symptomatic and preventive medication classifications

ATC code	Medication name	Category
A01AD11	Various	Symptomatic
A02AA10	Combinations	Symptomatic
A02AD01	Ordinary salt combinations	Symptomatic
A02AF02	Ordinary salt combinations and antiflatulents	Symptomatic
A02BA02	Ranitidine	Symptomatic
A02BA04	Nizatidine	Symptomatic
A02BC01	Omeprazole	Symptomatic
A02BC02	Pantoprazole	Symptomatic
A02BC03	Lansoprazole	Symptomatic
A02BC04	Rabeprazole	Symptomatic
A02BC05	Esomeprazole	Symptomatic
A02BX	Other drugs for peptic ulcer and gastro-oesophageal reflux disease (GORD)	Symptomatic
A03AA04	Mebeverine	Symptomatic
A03BB01	Butylscopolamine	Symptomatic
A03FA01	Metoclopramide	Symptomatic
A03FA03	Domperidone	Symptomatic
A04AA01	Ondansetron	Symptomatic
A06AA02	Docusate sodium	Symptomatic
A06AB02	Bisacodyl	Symptomatic
A06AB06	Senna glycosides	Symptomatic
A06AB08	Sodium picosulfate	Symptomatic
A06AB56	Senna glycosides, combinations	Symptomatic
A06AC	Bulk-forming laxatives	Symptomatic
A06AC01	Ispaghula (psylla seeds)	Symptomatic
A06AC03	Sterculia	Symptomatic
A06AC07	Triticum (wheat fibre)	Symptomatic
A06AD11	Lactulose	Symptomatic
A06AD15	Macrogol	Symptomatic
A06AD18	Sorbitol	Symptomatic
A07AA11	Rifaximin	Symptomatic
A07DA01	Diphenoxylate	Symptomatic
A07DA03	Loperamide	Symptomatic
A07EC01	Sulfasalazine	Symptomatic
A07EC02	Mesalazine	Symptomatic
A07FA51	Lactic acid producing organisms, combinations	Symptomatic
A09AA02	Multienzymes (lipase, protease etc.)	Symptomatic
A10AA02	Insulin isophane (human)	Preventive
A10AB05	Insulin aspart	Preventive
A10AC01	Insulin (human)	Preventive
A10AD01	Insulin (human)	Preventive
A10AD05	Insulin aspart	Preventive
A10AD06	Insulin degludec and insulin aspart	Preventive
A10AE04	Insulin glargine	Preventive
A10BA02	Metformin	Preventive
A10BB09	Gliclazide	Preventive
A10BB12	Glimepiride	Preventive
A10BD07	Metformin and sitagliptin	Preventive
A10BD08	Metformin and vildagliptin	Preventive
A10BD11	Metformin and linagliptin	Preventive
A10BD13	Metformin and alogliptin	Preventive
A10BD19	Linagliptin and empagliflozin	Preventive
A10BH01	Sitagliptin	Preventive
A10BH02	Vildagliptin	Preventive
A10BH03	Saxagliptin	Preventive
A10BH04	Alogliptin	Preventive
A10BH05	Linagliptin	Preventive
A10BX09	Dapagliflozin	Preventive

A10BX12	Empagliflozin	Preventive
A11A	Multivitamins, combinations	Preventive
A11B	Multivitamins, plain	Preventive
A11CC04	Calcitriol	Preventive
A11CC05	Colecalciferol	Preventive
A11DA01	Thiamine (vit B1)	Preventive
A11EB	Vitamin B-complex with vitamin C	Preventive
A11EX	Vitamin B-complex, other combinations	Preventive
A11GA01	Ascorbic acid (vit C)	Preventive
A11GB	Ascorbic acid (vitamin C), combinations	Preventive
A11HA02	Pyridoxine (vit B6)	Preventive
A11HA03	Tocopherol (vit E)	Preventive
A11JB	Vitamins with minerals	Preventive
A12AA	Calcium	Preventive
A12AA04	Calcium carbonate	Preventive
A12AA20	Calcium (different salts in combination)	Preventive
A12AX	Calcium, combinations with vitamin D and/or other drugs	Preventive
A12BA01	Potassium chloride	Preventive
A12CA01	Sodium chloride	Symptomatic
A12CB	Zinc	Preventive
A12CC30	Magnesium (different salts in combination)	Symptomatic
A12CD01	Sodium fluoride	Preventive
B01AA03	Warfarin	Preventive
B01AB05	Enoxaparin	Preventive
B01AC04	Clopidogrel	Preventive
B01AC06	Acetylsalicylic acid	Preventive
B01AC30	Combinations	Preventive
B01AE07	Dabigatran etexilate	Preventive
B01AF01	Rivaroxaban	Preventive
B01AF02	Apixaban	Preventive
B03AA02	Ferrous fumarate	Preventive
B03AA07	Ferrous sulfate	Preventive
B03AB05	Ferric oxide polymaltose complexes	Preventive
B03AC	Iron, parenteral preparations	Preventive
B03AD02	Ferrous fumarate	Preventive
B03AD03	Ferrous sulfate	Preventive
B03AE10	Various combinations	Preventive
B03BA01	Cyanocobalamin	Preventive
B03BA03	Hydroxocobalamin	Preventive
B03BB01	Folic acid	Preventive
B03XA02	Darbepoetin alfa	Symptomatic
B03XA03	Methoxy polyethylene glycol-epoetin beta	Symptomatic
B05CB01	Sodium chloride	Symptomatic
B05XA02	Sodium bicarbonate	Symptomatic
C01AA05	Digoxin	Preventive
C01BC04	Flecainide	Preventive
C01BD01	Amiodarone	Preventive
C01CA17	Midodrine	Preventive
C01DA02	Glyceryl trinitrate	Symptomatic
C01DA08	Isosorbide dinitrate	Symptomatic
C01DA14	Isosorbide mononitrate	Symptomatic
C01DX16	Nicorandil	Symptomatic
C02AB01	Methyldopa (levorotatory)	Preventive
C02AC05	Moxonidine	Preventive
C02CA01	Prazosin	Preventive
C03AA03	Hydrochlorothiazide	Preventive
C03BA04	Chlortalidone	Preventive
C03BA11	Indapamide	Preventive
C03CA01	Furosemide	Preventive
C03DA01	Spironolactone	Preventive

C05AA01	Hydrocortisone	Symptomatic
C05CA54	Troxerutin, combinations	Symptomatic
C07AA05	Propranolol	Preventive
C07AA07	Sotalol	Preventive
C07AB02	Metoprolol	Preventive
C07AB03	Atenolol	Preventive
C07AB07	Bisoprolol	Preventive
C07AB12	Nebivolol	Preventive
C07AG01	Labetalol	Preventive
C07AG02	Carvedilol	Preventive
C08CA01	Amlodipine	Preventive
C08CA02	Felodipine	Preventive
C08CA05	Nifedipine	Preventive
C08CA13	Lercanidipine	Preventive
C08DA01	Verapamil	Preventive
C08DB01	Diltiazem	Preventive
C08EX02	Perhexiline	Preventive
C09AA01	Captopril	Preventive
C09AA02	Enalapril	Preventive
C09AA04	Perindopril	Preventive
C09AA05	Ramipril	Preventive
C09AA06	Quinapril	Preventive
C09AA09	Fosinopril	Preventive
C09AA10	Trandolapril	Preventive
C09BA04	Perindopril and diuretics	Preventive
C09BA09	Fosinopril and diuretics	Preventive
C09BB02	Enalapril and lercanidipine	Preventive
C09BB04	Perindopril and amlodipine	Preventive
C09CA02	Eprosartan	Preventive
C09CA04	Irbesartan	Preventive
C09CA06	Candesartan	Preventive
C09CA07	Telmisartan	Preventive
C09CA08	Olmesartan medoxomil	Preventive
C09DA03	Valsartan and diuretics	Preventive
C09DA04	Irbesartan and diuretics	Preventive
C09DA06	Candesartan and diuretics	Preventive
C09DA07	Telmisartan and diuretics	Preventive
C09DA08	Olmesartan medoxomil and diuretics	Preventive
C09DB01	Valsartan and amlodipine	Preventive
C09DB02	Olmesartan medoxomil and amlodipine	Preventive
C09DB04	Telmisartan and amlodipine	Preventive
C09DX01	Valsartan, amlodipine and hydrochlorothiazide	Preventive
C09DX03	Olmesartan medoxomil, amlodipine and hydrochlorothiazide	Preventive
C10AA01	Simvastatin	Preventive
C10AA03	Pravastatin	Preventive
C10AA05	Atorvastatin	Preventive
C10AA07	Rosuvastatin	Preventive
C10AB04	Gemfibrozil	Preventive
C10AB05	Fenofibrate	Preventive
C10AC01	Colestyramine	Preventive
C10AX06	Omega-3-triglycerides incl. Other esters and acids	Preventive
C10AX09	Ezetimibe	Preventive
C10BA02	Simvastatin and ezetimibe	Preventive
C10BA05	Atorvastatin and ezetimibe	Preventive
C10BA06	Rosuvastatin and ezetimibe	Preventive
C10BX03	Atorvastatin and amlodipine	Preventive
D01AC01	Clotrimazole	Symptomatic
D01AC02	Miconazole	Symptomatic
D01AC08	Ketoconazole	Symptomatic
D01AE16	Amorolfine	Symptomatic

D02AB	Zinc products	Symptomatic
D02AC	Soft paraffin and fat products	Symptomatic
D02AE01	Carbamide	Symptomatic
D02AF	Salicylic acid preparations	Symptomatic
D02AX	Other emollients and protectives	Symptomatic
D04AB01	Lidocaine	Symptomatic
D06BA01	Silver sulfadiazine	Symptomatic
D06BX01	Metronidazole	Symptomatic
D07AA02	Hydrocortisone	Symptomatic
D07AB09	Triamcinolone	Symptomatic
D07AC01	Betamethasone	Symptomatic
D07AC13	Mometasone	Symptomatic
D07AC14	Methylprednisolone aceponate	Symptomatic
D07AD01	Clobetasol	Symptomatic
D07XA01	Hydrocortisone	Symptomatic
D08AG02	Povidone-iodine	Symptomatic
D08AJ01	Benzalkonium	Symptomatic
D11AX	Other dermatologicals	Symptomatic
D11AX18	Diclofenac	Symptomatic
G03CA03	Estradiol	Preventive
G03CA04	Estriol	Preventive
G03HA01	Cyproterone	Preventive
G04BD04	Oxybutynin	Symptomatic
G04BD08	Solifenacin	Symptomatic
G04BD12	Mirabegron	Symptomatic
G04BX	Other urologicals	Symptomatic
G04CA02	Tamsulosin	Symptomatic
G04CA04	Silodosin	Symptomatic
G04CA52	Tamsulosin and dutasteride	Symptomatic
G04CX	Other drugs used in benign prostatic hypertrophy	Symptomatic
H02AA02	Fludrocortisone	Symptomatic
H02AB02	Dexamethasone	Symptomatic
H02AB06	Prednisolone	Symptomatic
H02AB07	Prednisone	Symptomatic
H03AA01	Levothyroxine sodium	Preventive
H03BB01	Carbimazole	Preventive
J01AA02	Doxycycline	Symptomatic
J01DB01	Cefalexin	Symptomatic
J01EA01	Trimethoprim	Symptomatic
J01EE01	Sulfamethoxazole and trimethoprim	Symptomatic
J01FA06	Roxithromycin	Symptomatic
J01FA10	Azithromycin	Symptomatic
J01XE01	Nitrofurantoin	Symptomatic
J01XX	Other antibacterials	Symptomatic
J01XX05	Methenamine	Symptomatic
L01BA01	Methotrexate	Preventive
L01XX05	Hydroxycarbamide	Preventive
L02AB02	Medroxyprogesterone	Preventive
L02AE02	Leuprorelin	Preventive
L02AE03	Goserelin	Preventive
L02BA01	Tamoxifen	Preventive
L02BB03	Bicalutamide	Preventive
L02BG04	Letrozole	Preventive
L04AA01	Cyclosporin	Preventive
L04AA13	Leflunomide	Preventive
L04AA27	Fingolimod	Preventive
L04AX01	Azathioprine	Preventive
M01AC06	Meloxicam	Symptomatic
M01AE01	Ibuprofen	Symptomatic
M01AH01	Celecoxib	Symptomatic

M01AX05	Glucosamine	Symptomatic
M01CA	Quinolines	Symptomatic
M02AC	Preparations with salicylic acid derivatives	Symptomatic
M02AX10	Various	Symptomatic
M03BX01	Baclofen	Symptomatic
M04AA01	Allopurinol	Preventive
M04AC01	Colchicine	Symptomatic
M05BA04	Alendronic acid	Preventive
M05BA07	Risedronic acid	Preventive
M05BB03	Alendronic acid and colecalciferol	Preventive
M05BB05	Alendronic acid, calcium and colecalciferol, sequential	Preventive
M05BX04	Denosumab	Preventive
N02AA	Natural opium alkaloids	Symptomatic
N02AA01	Morphine	Symptomatic
N02AA03	Hydromorphone	Symptomatic
N02AA05	Oxycodone	Symptomatic
N02AA55	Oxycodone and naloxone	Symptomatic
N02AB03	Fentanyl	Symptomatic
N02AE01	Buprenorphine	Symptomatic
N02AJ06	Codeine and paracetamol	Symptomatic
N02AX02	Tramadol	Symptomatic
N02AX06	Tapentadol	Symptomatic
N02BE01	Paracetamol	Symptomatic
N02CX01	Pizotifen	Preventive
N03AA03	Primidone	Preventive
N03AB02	Phenytoin	Preventive
N03AE01	Clonazepam	Symptomatic
N03AF01	Carbamazepine	Preventive
N03AG01	Valproic acid	Preventive
N03AX09	Lamotrigine	Preventive
N03AX11	Topiramate	Preventive
N03AX12	Gabapentin	Symptomatic
N03AX14	Levetiracetam	Preventive
N03AX16	Pregabalin	Symptomatic
N04BA02	Levodopa and decarboxylase inhibitor	Symptomatic
N04BA03	Levodopa, decarboxylase inhibitor and COMT inhibitor	Symptomatic
N04BB01	Amantadine	Symptomatic
N04BC05	Pramipexole	Symptomatic
N04BC06	Cabergoline	Symptomatic
N04BC09	Rotigotine	Symptomatic
N04BD02	Rasagiline	Symptomatic
N04BX02	Entacapone	Symptomatic
N05AB04	Prochlorperazine	Symptomatic
N05AH03	Olanzapine	Symptomatic
N05AH04	Quetiapine	Symptomatic
N05AN01	Lithium	Symptomatic
N05AX08	Risperidone	Symptomatic
N05BA01	Diazepam	Symptomatic
N05BA04	Oxazepam	Symptomatic
N05BA08	Bromazepam	Symptomatic
N05BA12	Alprazolam	Symptomatic
N05CD02	Nitrazepam	Symptomatic
N05CD07	Temazepam	Symptomatic
N05CF01	Zopiclone	Symptomatic
N05CH01	Melatonin	Symptomatic
N05CM19	Suvorexant	Symptomatic
N06AA09	Amitriptyline	Symptomatic
N06AA10	Nortriptyline	Symptomatic
N06AA12	Doxepin	Symptomatic
N06AA16	Dosulepin	Symptomatic

N06AB03	Fluoxetine	Preventive
N06AB04	Citalopram	Preventive
N06AB05	Paroxetine	Preventive
N06AB06	Sertraline	Preventive
N06AB08	Fluvoxamine	Preventive
N06AB10	Escitalopram	Preventive
N06AG02	Moclobemide	Preventive
N06AX03	Mianserin	Preventive
N06AX11	Mirtazapine	Preventive
N06AX16	Venlafaxine	Preventive
N06AX18	Reboxetine	Preventive
N06AX21	Duloxetine	Preventive
N06AX23	Desvenlafaxine	Preventive
N06DA02	Donepezil	Preventive
N06DA03	Rivastigmine	Preventive
N06DA04	Galantamine	Preventive
N06DX01	Memantine	Preventive
N07AA02	Pyridostigmine	Symptomatic
N07AB02	Bethanechol	Symptomatic
N07BC01	Buprenorphine	Symptomatic
N07CA01	Betahistine	Symptomatic
N07XX06	Tetrabenazine	Symptomatic
P01BC01	Quinine	Symptomatic
R01AD05	Budesonide	Symptomatic
R02AD01	Benzocaine	Symptomatic
R03AC02	Salbutamol	Symptomatic
R03AC03	Terbutaline	Symptomatic
R03AC12	Salmeterol	Preventive
R03AC13	Formoterol	Preventive
R03AC18	Indacaterol	Preventive
R03AK06	Salmeterol and fluticasone	Preventive
R03AK07	Formoterol and budesonide	Preventive
R03AK10	Vilanterol and fluticasone furoate	Preventive
R03AK11	Formoterol and fluticasone	Preventive
R03AL06	Olodaterol and tiotropium bromide	Preventive
R03AL08	Vilanterol, umeclidinium bromide and fluticasone furoate	Preventive
R03BA01	Beclometasone	Preventive
R03BA02	Budesonide	Preventive
R03BA05	Fluticasone	Preventive
R03BA08	Ciclesonide	Preventive
R03BB01	Ipratropium bromide	Symptomatic
R03BB04	Tiotropium bromide	Preventive
R03BB05	Aclidinium bromide	Preventive
R03BB06	Glycopyrronium bromide	Preventive
R03BB07	Umeclidinium bromide	Preventive
R03DA04	Theophylline	Preventive
R05CA10	Combinations	Symptomatic
R05CB02	Bromhexine	Symptomatic
R05X	Other cold preparations	Symptomatic
R06AD02	Promethazine	Symptomatic
R06AE07	Cetirizine	Symptomatic
R06AX13	Loratadine	Symptomatic
R06AX26	Fexofenadine	Symptomatic
S01AA12	Tobramycin	Symptomatic
S01AD03	Aciclovir	Symptomatic
S01BA01	Dexamethasone	Symptomatic
S01BA07	Fluorometholone	Symptomatic
S01BC03	Diclofenac	Symptomatic
S01EA	Sympathomimetics in glaucoma therapy1)	Preventive
S01EA05	Brimonidine	Preventive

S01EB01	Pilocarpine	Preventive
S01EC	Carbonic anhydrase inhibitors	Preventive
S01EC01	Acetazolamide	Preventive
S01EC04	Brinzolamide	Preventive
S01EC54	Brinzolamide, combinations	Preventive
S01ED01	Timolol	Preventive
S01ED51	Timolol, combinations	Preventive
S01EE01	Latanoprost	Preventive
S01EE03	Bimatoprost	Preventive
S01EE04	Travoprost	Preventive
S01EE05	Tafluprost	Preventive
S01FA01	Atropine	Symptomatic
S01X	Other ophthalmologicals	Symptomatic
S01XA20	Artificial tears and other indifferent preparations	Symptomatic
S02AA01	Chloramphenicol	Symptomatic
S02DC	Indifferent preparations	Symptomatic
V03AE01	Polystyrene sulfonate	Symptomatic
V03AN01	Oxygen	Symptomatic
V06DB	Fat/carbohydrates/proteins/minerals/vitamins, combinations	Preventive
V06DD	Amino acids, incl. Combinations with polypeptides	Preventive
V06DE	Amino acids/carbohydrates/minerals/vitamins, combinations	Preventive
V06DX	Other combinations of nutrients	Preventive

Supplementary Table 3a. Mean number of symptomatic and preventive medications according to age and frailty

	Frailty status				Age (years)			
	**Non-frail**	**Frail**	**Most-frail**	**p-value**	**<80**	**80-89**	**≥90**	**p-value**
**Australia (n=550)**								
**No. of medications, mean (SD)**	8.9 (3.6)	9.6 (4.0)	10.1 (4.0)	0.0719	10.3 (4.5)	10.2 (4.1)	9.2 (3.7)	**0.0134**
Symptomatic	3.4 (2.6)	4.0 (2.6)	4.8 (2.6)	**<0.0001**	5.0 (2.9)	4.4 (2.6)	3.9 (2.5)	**0.0042**
Preventive	5.5 (2.3)	5.7 (2.6)	5.4 (2.6)	0.3896	5.3 (2.8)	5.8 (2.6)	5.3 (2.4)	0.0781
**Japan (n=333)**								
**No. of medications, mean (SD)**	7.4 (3.7)	7.5 (3.6)	7.8 (3.7)	0.6924	8.4 (4.8)	7.7 (3.6)	7.4 (3.1)	0.3456
Symptomatic	4.5 (2.7)	4.7 (2.8)	4.8 (2.5)	0.8614	5.2 (3.3)	4.7 (2.6)	4.5 (2.3)	0.3554
Preventive	2.9 (1.8)	2.8 (2.0)	3.1 (2.4)	0.6165	3.2 (2.7)	3.0 (2.4)	2.9 (1.9)	0.7815

Note: One-way analysis of variance (ANOVA)

Supplementary Table 3b. Sensitivity analysis excluding Japanese residents who died within three months of baseline data collection

	Frailty status				Age (years)			
	**Non-frail**	**Frail**	**Most-frail**	**p-value**	**<80**	**80-89**	**≥90**	**p-value**
**Japan (n=323)**								
**No. of medications, mean (SD)**	7.5 (3.7)	7.5 (3.6)	7.7 (3.7)	0.8640	8.4 (4.8)	7.7 (3.7)	7.3 (3.0)	0.2370
Symptomatic	4.5 (2.7)	4.7 (2.8)	4.7 (2.5)	0.9496	5.2 (3.3)	4.7 (2.6)	4.4 (2.3)	0.2751
Preventive	3.0 (1.8)	2.8 (2.0)	3.1 (2.4)	0.6724	3.2 (2.7)	3.0 (2.3)	2.9 (1.8)	0.6721

Note: One-way analysis of variance (ANOVA)

Supplementary Table 4. List of Anatomical Therapeutic Chemical (ATC) codes for medication classes

Medication class	ATC code
Anticholinesterase	N06DA
Antiplatelet	B01AC
Angiotensin II receptor blocker (ARB)	C09CA
Benzodiazepine	N05BA
Beta-blocker	C07AB
Buprenorphine patch	N02AE
Contact laxative	A06AB
Dihydropyridine calcium channel blocker (CCB)	C08CA
Direct factor Xa inhibitor	B01AF
Gastrointestinal (GI) stimulant	A03FA
Loop diuretic	C03CA
Ocular lubricant	S01XA
Osmotic laxative	A06AD
Paracetamol	N02BE
Potassium	A12BA
Proton pump inhibitor (PPI)	A02BC
Pregabalin & derivatives (gabapentin, levetiracetam, lamotrigine, topiramate)	N03AX
Selective noradrenaline reuptake inhibitor (SNRI) & mirtazapine	N06AX
Selective serotonin reuptake inhibitor (SSRI)	N06AB
Statin	C10AA
Thyroid hormone	H03AA
Topical heparinoid	C05BA
Valproate	N03AG
Vitamin B12	B03BA
Vitamin D & analogues	A11CC
Xanthine oxidase inhibitor	M04AA
Z-drug	N05CF

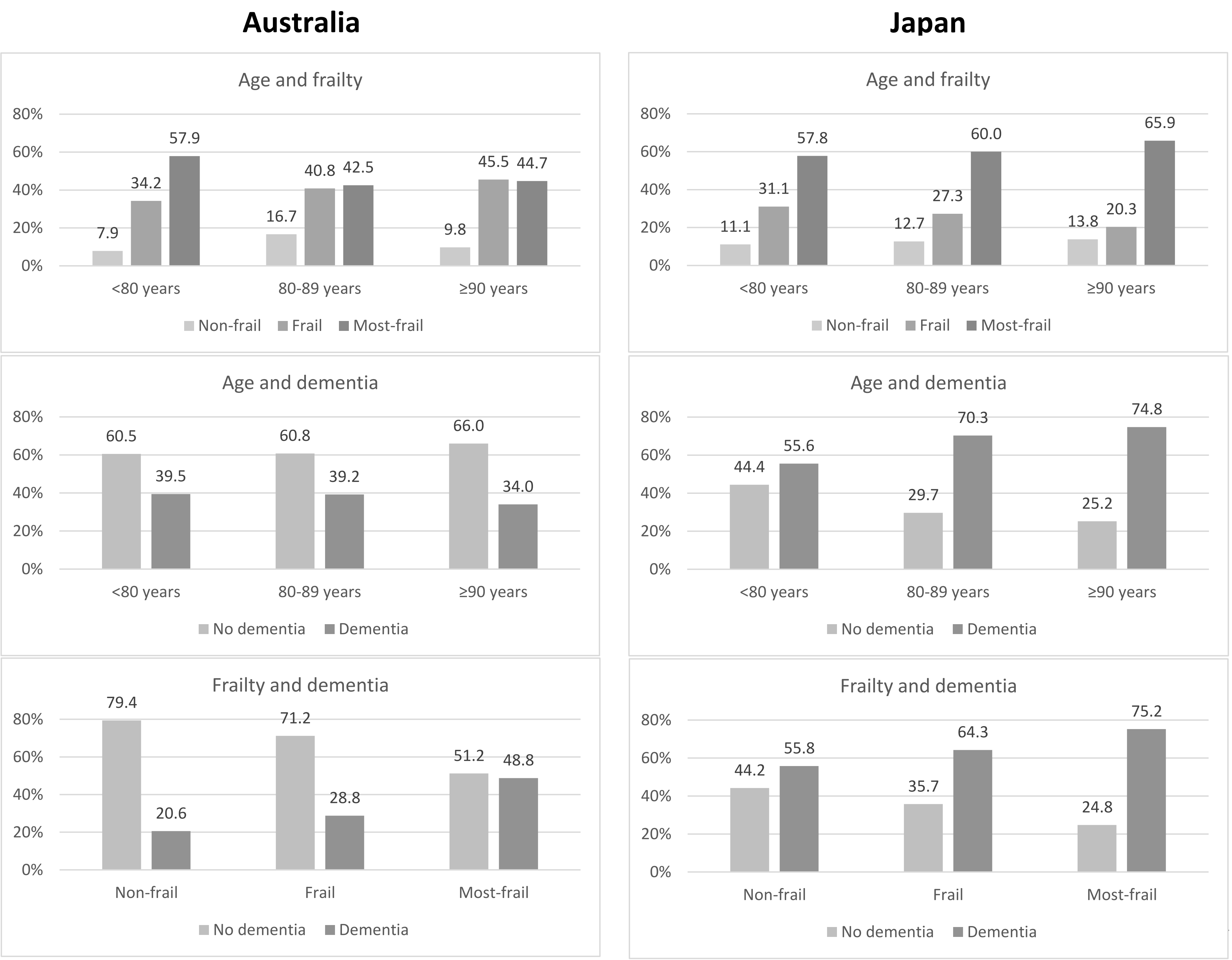

Supplementary Figure 1. Proportion of residents stratified by age, frailty, and dementia status
